# A Baby With Complete Androgen Insensitivity Syndrome and the Fortuitous Discovery of 45,X/46,XY Mosaicism

**DOI:** 10.7759/cureus.43352

**Published:** 2023-08-11

**Authors:** Wai Yu Wong, Lap Ming Wong, Yuk Him Tam, Ho Ming Luk

**Affiliations:** 1 Paediatrics and Adolescent Medicine, Tuen Mun Hospital, Hong Kong, HKG; 2 Paediatric Surgery, Hong Kong Children's Hospital, Hong Kong, HKG; 3 Clinical Genetics Service Unit, Hong Kong Children's Hospital, Hong Kong, HKG

**Keywords:** gonadal malignancy, turner mosaicism, gonadal dysgenesis, complete androgen insensitivity syndrome, differences of sex development

## Abstract

Disorders of sex development (DSD) are caused by defects in the complex sexual differentiation cascade, resulting in discordance among an individual's genetic, gonadal, and genital sexes. It affects one in 4,500 live births. A wide spectrum of genital phenotypes can be found depending on the underlying pathogenic mechanism and the developmental stage that is affected. We herein report a newborn with female external genitalia but palpable gonads at labia majora with normal testicular function and structure, which is typical of complete androgen insensitivity syndrome (CAIS). The genetic study revealed 45,X/46,XY mosaicism and c.2081A>C missense androgen receptor gene mutation, indicating the likelihood of co-existing CAIS. This case demonstrated the importance of correlating genital phenotype and the underlying pathogenic mechanism, to provide appropriate management of DSD. Important considerations on managing the gonads about the risks of gonadal malignancies are also discussed.

## Introduction

Genetic sex is primarily determined by the sex chromosomes of an individual at fertilization. However, sexual differentiation involves a complex cascade of events during embryonic development where the sexually indifferent gonads develop into male or female sexual characteristics with corresponding internal and external genitalia [1]. At the stage of sexual determination, the primordial gonads develop into testes in the presence of the *SRY* gene on the Y chromosome or develop into ovaries in the absence of the gene. Subsequently, the internal and external genitalia develop into male morphologies in the presence of testicular hormones and into female morphologies otherwise. Disorders in any of these events in the sexual differentiation cascade can cause discordance of the genetic, gonadal, and genital sexes of an individual, collectively described as disorders of sex development (DSD) [1].

DSD affects approximately one in 4,500 live births [2]. A wide spectrum of genital phenotypes and clinical presentation can be found as a result, depending on the underlying pathogenic mechanism and the stage of development affected.

We herein report a baby affected by 45,X/46,XY mosaicism as well as an androgen receptor (AR) gene mutation, who presented with complete androgen insensitivity syndrome (CAIS). We demonstrate the importance of correlating genital phenotype and the underlying pathogenic mechanism, to provide appropriate management of DSD.

## Case presentation

Baby Y was born at 39 weeks of gestation by normal spontaneous vaginal delivery, with a birth weight of 3.52 kg. A noninvasive prenatal test of baby Y (SafeT21) was arranged in the first trimester to screen for trisomies, and it showed 46,XY karyotype. However, subsequent serial antenatal ultrasonography demonstrated undervirilization of the external genitalia. As a result, amniocentesis was performed at 21 weeks of gestation and similarly demonstrated 46,XY karyotype.

**Figure 1:**
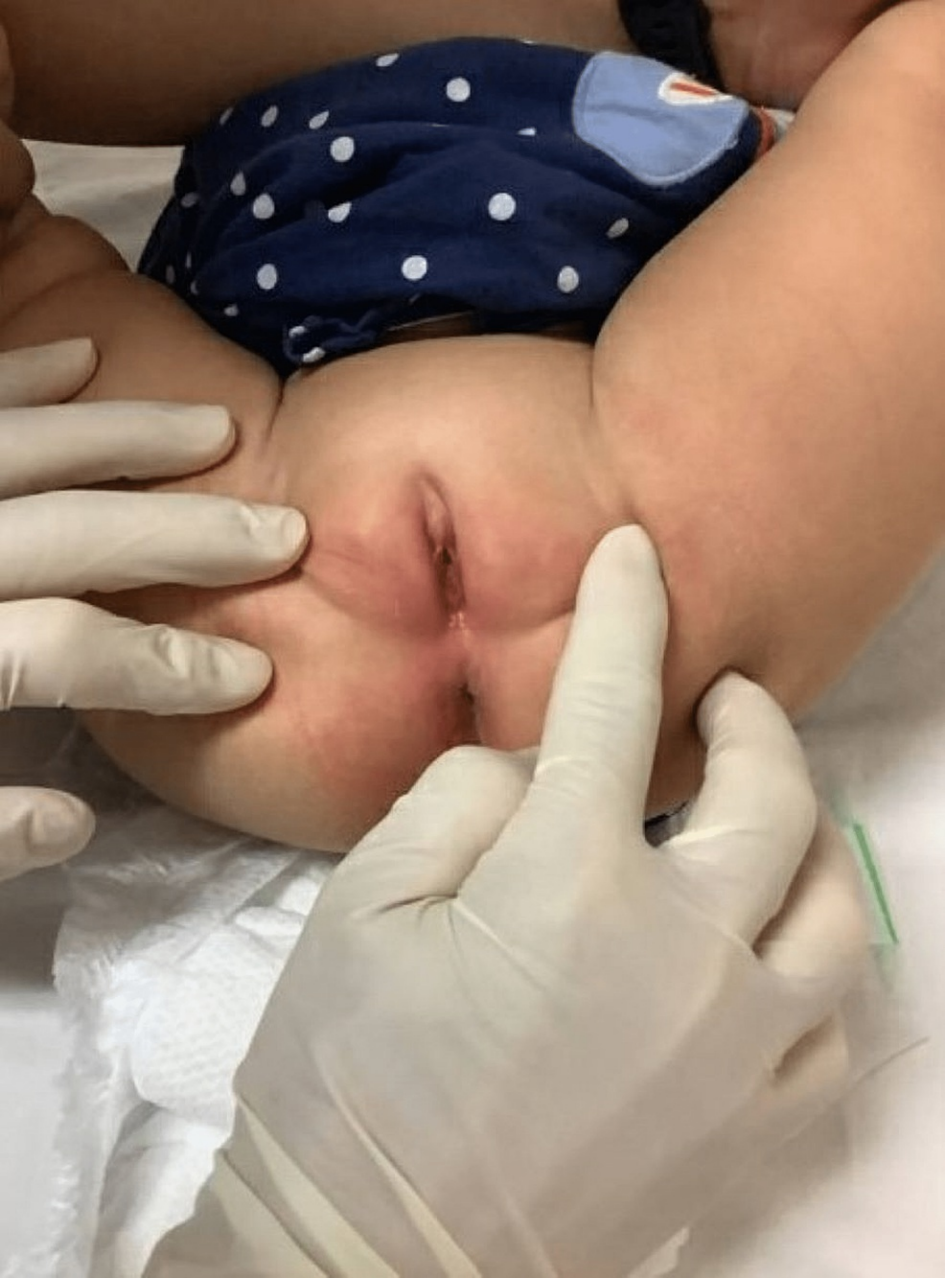
External genitalia of baby Y. This figure demonstrated unfused labioscrotal folds, impalpable phallus, urethral meatus at the perineum, and separate urogenital openings. This figure shows female external genitalia without any sexual ambiguity. The labioscrotal folds were full-looking, with normal infantile-sized gonads.

Postnatal examination of the external genitalia revealed unfused labioscrotal folds with normal pigmentation and absence of rugae. Gonads were identified at the upper labial folds without a palpable phallus. Urethral meatus was found at the perineum, with a separate vaginal opening, and the patient's anus was identified at the normal anatomical position. Overall, the external genitalia were typical of a female phenotype (Figure 1). The External Genitalia Score (EGS) was 3 [3], as the scores of labioscrotal fusion, genital tubercle length, and urethral opening position were zero, while the score of gonad was 1.5 on each side (Table 1). Given the palpable gonads of normal infantile size associated with undervirilized external genitalia, the working diagnosis was 46,XY DSD.

**Table 1:** External genitalia score. Source: [3]. GT, genital tubercle

Score	Labioscrotal fusion	GT length (mm)	Urethral opening	Left gonad	Right gonad
3.0	Fused	>31	Top of GT		
2.5		26-30	Coronal glandular		
2.0			Along GT		
1.5	Posterior fusion	21-25	At base of GT	Labioscrotal	Labioscrotal
1.0		10-20	Labioscrotal	Inguinal labioscrotal	Inguinal labioscrotal
0.5				Inguinal	Inguinal
0.0	Unfused	<10	Perineal	Impalpable	Impalpable

Further investigations were carried out to ascertain the diagnosis (Tables 2-3). First, to evaluate the hypothalamic-pituitary-gonadal axis, the luteinizing hormone (LH) level, the follicle-stimulating hormone (FSH) level, and estradiol and testosterone levels were measured on day five of life, showing appropriate levels for the male gender during the neonatal period. At one month of life, an LH-releasing hormone (LHRH) stimulation test was performed. The peak LH level was 5.3 IU/L, while the peak FSH level was 3.0 IU/L, representing a good response at mini-puberty, with no evidence of hypogonadotropic hypogonadism.

**Table 2:** Biochemical profile of baby Y. LH, luteinizing hormone; FSH, follicle-stimulating hormone; DHEAS, dehydroepiandrosterone sulfate; LHRH, luteinizing hormone releasing hormone; AMH, anti-Mullerian hormone; F, female; M, male

Age (days of life)	Test		Result	Reference
5	LH		1.1 IU/L	M: 0.2-3.8 IU/L; F: < 2.4 IU/L
	FSH		1.3 IU/L	N/A below 30 days of life
	Testosterone		1.43 nmol/L	M: <6.5 nmol/L; F: <0.8 nmol/L
	Oestradiol		49 pmol/L	N/A below 15 days of life
	Cortisol		285 nmol/L	171-536 nmol/L
	Sodium		137 mmol/L	133-146 mmol/L
	Potassium		5.2 mmol/L	3.7-5.9 mmol/L
	Androstenedione		0.7 nmol/L	<2.5 nmol/L
	DHEAS		7.1 umol/L	2.9-16.5 umol/L
35	LHRH stimulation test	Peak LH	5.3 IU/L	
		Peak FSH	3.1 IU/L	
	AMH		1,335 pmol/L	M: 287-1,242 pmol/L; F: 0.5-38.58 pmol/L

**Table 3:** Prolonged HCG stimulation test. HCG, human chorionic gonadotropin; N/A, not available

Prolonged HCG stimulation test	Day 1	Day 4	Day 21
Testosterone	<0.5 nmol/L	23.2 nmol/L	44.6 nmol/L
Androstenedione	1.4 nmol/L	1.4 nmol/L	2.7 nmol/L
Testosterone:androstenedione	N/A	16.5	16.5

Second, to evaluate the testicular function, anti-Mullerian hormone (AMH) was measured to assess Sertoli cell function, while a prolonged human chorionic gonadotropin (HCG) stimulation test was performed to assess Leydig cell function. AMH was slightly above the male infant range. Adequate rise of testosterone demonstrated normal Leydig cell function. This result has ruled out Swyer syndrome as a cause of discordance of phenotypic sex and karyotype.

The presence of normal electrolytes, cortisol, testosterone, and precursor levels indicated a lower likelihood of adrenal steroidogenesis defects. At prolonged HCG stimulation test, the testosterone:androstenedione (T:A) ratio remained high after stimulation, which excluded 17-beta hydroxysteroid dehydrogenase 3 deficiency that typically has a T:A ratio <0.8. Urine steroid profile after HCG stimulation also showed no evidence of atypical congenital adrenal hyperplasia (CAH) leading to undervirilization of males, such as steroidogenic acute regulatory (StAR) deficiency or P450 oxidoreductase deficiency.

At three months of age, the urine steroid profiling showed a normal ratio of 5-alpha to 5-beta reduced steroid metabolites, ruling out the possibility of 5-alpha reductase 2 deficiency.

For imaging evaluation of internal genital organs, ultrasonography of the pelvis was performed on day six of life and identified testes at bilateral lower inguinal canals with comparable sizes and normal parenchymal echogenicity. The vaginal canal was present, while the uterus and ovaries could not be detected, reflecting Mullerian regression and adequate action of AMH. Wolffian derivatives were absent. The urinary system was normal.

**Figure 2:**
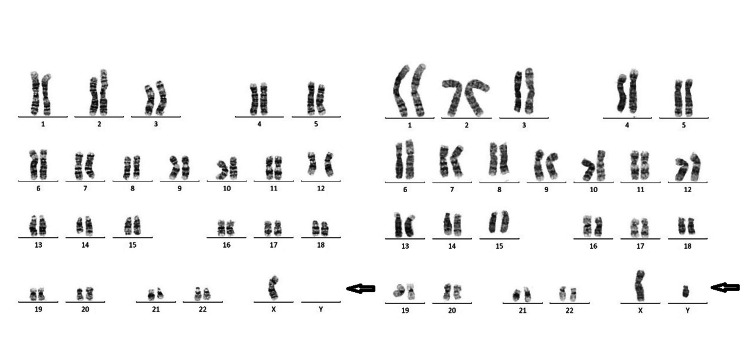
Karyotype of baby Y. This illustration depicts a karyotype at a resolution of 400-550 bands, illustrating a mosaic pattern of 45,X (4) and 46,XY (46) cells.

To our surprise, karyotyping on 50 leukocytes revealed a mosaic pattern with one cell line of four cells showing one X chromosome, and a second cell line of 46 cells with a normal diploid male pattern of one X chromosome and one Y chromosome, that is, 45,X(4)/46,XY(46) mosaicism was found in this patient (Figure 2).

As the patient had bilateral testes of normal infantile size and normal testicular function, the extent of undervirilization could not be explained by the abnormal karyotype alone. Therefore, further genetic testing for AR mutation was performed. A hemizygous missense c.2081A>C variant was detected in the AR gene, which was classified as a variant of unknown significance. The same hemizygous mutation was also detected in the mother. The nine-year-old elder sister had normal female genitalia and 46,XX karyotype. The seven-year-old elder brother had normal male genitalia and 46,XY karyotype. AR mutation was not detected in both siblings.

The baby was assigned to be reared as a female after a thorough multidisciplinary discussion with the family. Prophylactic gonadectomy in early childhood was considered to clarify the nature of gonads and eliminate the risk of future malignancy.

## Discussion

Fortuitous finding of 45,X/46,XY mosaicism in a patient with CAIS

45,X/46,XY mosaicism is a chromosomal disorder of sexual differentiation, which affects approximately 1.5 in 10,000 live births [4,5]. Affected individuals exhibit a wide variety of genital phenotypes, from females with Turner’s features, to children with ambiguous genitalia, to phenotypically normal males [6]. Most of the larger case series did not find a correlation between the proportion of mosaicism in blood and gonadal karyotype, nor the degree of phenotypic abnormality [5-8]. Rather, the relative proportion of the two cell lines in gonads determines the degree of virilization [9,10]. A variety of gonads may be found in these patients, including streak gonads, streak ovaries, dysgenetic testes, normal testes, and in some cases, gonadoblastomas [5-11]. 

A testicularized gonadal phenotype with greater Sertoli cell and Leydig cell function is associated with a virilized genital phenotype, while a less differentiated gonad is more likely associated with nonvirilized genitalia [12]. The studies by Huang et al. [13], Telvi et al. [7], and Matsumoto et al. [6] had the consistent observations that individuals with 45,X/46,XY karyotype and female phenotype most likely have intra-abdominal streak gonads or streak ovaries. Those with palpable scrotal or inguinal gonads develop male external genitalia. With unilateral or bilateral dysgenetic gonads, the individuals have variable degrees of virilization.

In baby Y, with inguinal gonads of normal size and echogenicity, normal baseline and HCG-stimulated testosterone level, normal AMH level, and absence of Mullerian structures on ultrasonography, her gonads were most likely testes with normal Leydig and Sertoli cell function, although gonadal biopsy or gonadectomy was not yet performed to elucidate the gonadal histology.

Androgen insensitivity syndrome (AIS) is an X-linked recessive genetic disorder caused by mutations in the AR gene located in the Xq11-q12 region. Affected individuals manifest a variable degree of androgen insensitivity and are clinically classified into complete, partial, and mild AIS [14]. CAIS affects one in 20,400 to 99,100 chromosomal males [15,16]. A total of 1,029 mutations in the AR gene has been reported in the Androgen Receptor Gene Mutation Database in 2012 [17].

For the genital phenotype, CAIS patients typically have normal testes found in the intra-abdominal cavity, inner inguinal ring, or labia majora. In the absence of testosterone responsiveness at the target tissue, Wolffian structures do not develop. Owing to the normal secretion of AMH by Sertoli cells, Mullerian structures regress and lead to the absence of a uterus and fallopian tubes. They have female external genitalia with blind-ending vaginas of variable length [18,19].

In baby Y, a hemizygous missense c.2081A>C variant was detected, which leads to the substitution of the 694th codon from glutamine to proline, in the ligand-binding domain (LBD) region of the AR protein. The mutation has not been documented in databases, including ClinVar, the Human Gene Mutation Database, the Single Nucleotide Polymorphism Database, the Genome Aggregation Database, as well as the Androgen Receptor Gene Mutations Database [17]. It was, however, classified as *likely pathogenic* in the Varsome search engine.

From the aforementioned discussion, baby Y had the typical clinical and biochemical features of CAIS. The 45,X/46,XY mosaicism was likely a fortuitous finding, which produced no effect on the genital phenotype at the current infantile stage. In a study by Hornig et al. [20], in six of the 15 individuals with 45,X/46,XY mosaicism, reduced AR mRNA expression and AR activity were found in genital skin fibroblasts compared with 46,XY controls, which correlated with the degree of undervirilization. However, they did not detect any mutation in the coding region of the AR gene. Concomitant finding of 45,X/46,XY mosaicism and AR mutation, as in baby Y, is a novel finding that has not been previously reported.

It was observed that phenotypic males with 45,X/46,XY karyotypes have a strong chance of testicular function adequate for spontaneous pubertal onset with variable needs of subsequent testosterone replacement [21]. Although baby Y was undervirilized due to concomitant AR mutation, she is likely to produce adequate androgens that allow spontaneous pubertal onset if the testes are preserved. Baby Y is expected to have normal breast development, absent or very sparse pubic and axillary hair growth, and primary amenorrhea at puberty, as a typical CAIS patient [18]. Longitudinal growth is commonly impaired in 45,X/46,XY individuals, and they may benefit from growth hormone treatment [9,21]. It was suggested that all 45,X/46,XY children should undergo evaluation for cardiac malformations, renal abnormalities, autoimmune conditions, otitis media, hearing deficits, learning disability, and behavioral problems [9].

Malignancy risk and gonadal management

Management of DSD includes the assignment of sex of rearing, the decision of gonadectomy and its timing, hormonal replacement, surveillance of tumor risk, fertility management, as well as psychosocial aspects of gender identity and sexual health. Nowadays, patients’ autonomy and shared decision-making are advocated, especially concerning irreversible genital surgeries. By postponing gonadectomy, patients are allowed to make informed decisions in their management and may benefit from endogenous sex hormone production in the progression of pubertal development. This has to be balanced against the risk of gonadal malignancy.

The estimated prevalence of testicular germ cell tumors in patients with AIS was 5.5%, while that for CAIS was 0.8% [22]. Various studies have shown a low risk of malignancy in prepubertal patients. In a study by Ahmed et al. [23], prophylactic gonadectomy was performed in 81 patients with CAIS, mostly before 18 years of age, and found no malignant lesions. In another study by Hannema et al. [24], prophylactic gonadectomy was performed in 44 patients with CAIS, revealing carcinoma in situ in two patients only, aged 17 and 53 years, respectively. The youngest reported case of malignant tumor in CAIS was aged 14 years at diagnosis [25]. With this evidence, the current recommendation supports delaying gonadectomy until postpubertal age to allow spontaneous completion of puberty under the effect of estradiol from peripheral aromatization of testosterone produced by the retained testes. Patients are allowed to take part in their medical decision-making at a more mature age too [14,18,19]. Surveillance can be done by interval ultrasonography of inguinal or labioscrotal gonads and magnetic resonance imaging of intra-abdominal gonads. Gonadal biopsy and immunohistochemical staining of markers, such as OCT3/4, can be helpful to stratify the risk of malignancy and guide the decision on gonadectomy [18,19].

As for 45,X/46,XY mosaicism and its variants, gonadal tumor risk was estimated to be 15% to 40%, significantly higher than 46,XY DSD caused by defective androgen production or action [22]. In a study of 48 patients with 45,X/46,XY karyotype by Cools et al. [26], the risk of tumors and preneoplastic lesions were 13%, 52%, and 2.2%, respectively, in the mild undervirilization, ambiguous genitalia, and female genotype group. The author proposed that tumor risk is most pronounced in immature or poorly differentiated gonadal tissues, and the degree of testicularization of gonads can be reflected by their phenotypes. In contrast to their findings, other studies have reported a high malignancy risk in the 45,X/46,XY patients with female phenotype. Matsumoto et al. reported a malignancy risk of 67% (6/9) in 45,X/46,XY individuals with female phenotype [6]. In smaller studies by Huang et al. [13], Tam et al. [27], and Coyle et al. [10], a high risk of malignancy in patients with female genitalia was described, with a rate of 13% (2/16), 55% (6/11), and 36% (4/11), respectively. The Consensus Statement on Management of Intersex Disorders suggested early removal of streak gonads in male patients with mixed gonadal dysgenesis and early bilateral gonadectomy in females with gonadal dysgenesis with Y-chromosomes. Scrotal testes in patients with gonadal dysgenesis are also at a higher risk of malignant transformation and should be biopsied at puberty [28]. Weidler et al. and Cools et al. suggested elective gonadectomy for those with female phenotype, a low threshold for gonadectomy for those with ambiguous genitalia, and orchidopexy of testes to an easily palpable location, regular surveillance by clinical examination and imaging, and gonadal biopsy to look out for premalignant and malignant lesions for those with normal or mildly undervirilized male phenotype [26,29]. Similarly, Wolffenbuttel et al. suggested relocation of abdominal gonads to a more superficial position and simultaneous bilateral gonadal biopsy. Immunohistochemical staining, such as TSPY and OCT3/4, can help to stratify the risk of malignancy and guide the decision of gonadal management [30].

The assessment of gonadal tumor risk and, consequently, the determination of gonadal management, is less obvious in baby Y. The clinical presentation of 45,X/46,XY mosaicism is complicated by the concomitant CAIS. Baby Y has gonads at the labial folds with normal testicular features on ultrasonography with normal androgen and AMH production, suggesting that they have appropriate testicular differentiation with a low risk of malignant transformation. However, this cannot be reflected by the degree of virilization of external genitalia because of concomitant CAIS. The family was taken through a thorough discussion, and a joint decision was made to rear the baby as a female and to consider prophylactic gonadectomy in early childhood to clarify the nature of gonads and eliminate the risk of future malignancy. The risk of surgery under general anesthesia, the need of lifelong hormonal replacement, and the implications on fertility were discussed with the family.

## Conclusions

We have presented a baby with typical clinical presentation of complete androgen insensitivity syndrome, i.e. external genitalia of female phenotype but palpable gonads of good size at labia majora with normal testicular hormonal secretion and normal structure on ultrasonography. Subsequent karyotyping revealed 45,X/46,XY mosaicism and genetic test showed a hemizygous missense mutation at androgen receptor gene in the LBD region. This case demonstrated the importance of identifying the underlying cause of DSD to explain the clinical phenotype. The underlying diagnosis affects the approach of management, especially the decision on gonadectomy based on the risk of malignant transformation.
